# Telerehabilitation and Physical Therapy: Proposal for a Therapeutic Assessment Applied to Vestibular Dysfunctions

**DOI:** 10.1055/s-0045-1810116

**Published:** 2025-10-16

**Authors:** Lucas Barbosa de Araújo, Karla Vanessa Rodrigues Soares Menezes, Jully Israely de Azevedo Rodolfo, Maria das Graças de Araújo Lira, Karyna Myrelly Oliveira Bezerra de Figueiredo Ribeiro

**Affiliations:** 1 Department of Physical Therapy, Post-Graduate Program in Physical Therapy, Universidade Federal do Rio Grande do Norte, Natal, RN, Brazil; 2 School of Rehabilitation, Université de Montréal, Montréal, QC, Canada; 3 Department of Physical Therapy, Universidade Federal do Rio Grande do Norte, Natal, RN, Brazil

**Keywords:** dizziness, health evaluation, telehealth, vertigo, vestibular diseases

## Abstract

**Introduction:**

Telerehabilitation has been used in several areas of physical therapy, including for respiratory, neurological, and musculoskeletal functions of patients with coronavirus disease 2019 (COVID-19), after stroke, and after hospital discharge (respectively). However, a few studies investigated protocols for assessing vestibular dysfunctions using teleconsultation.

**Objective:**

To propose a protocol for remote physical therapy assessment of vestibular dysfunctions.

**Methods:**

A literature review on telerehabilitation in physical therapy was conducted in the PubMed and SciELO databases using the search terms *telehealth* , *telerehabilitation* , *vestibular disease* , *dizziness* , *vertigo* , and *postural balance* . Four physical therapists with experience in the vestibular rehabilitation field discussed the collected data and suggested adaptations for remote clinical and functional tests to assess patients with vestibular dysfunctions.

**Results:**

The proposed protocol for remote assessment of vestibular dysfunctions comprised anamnesis, adaptations of nine oculomotors, two static balance, and one dynamic gait balance tests, a questionnaire assessing the impact of dizziness on quality of life, and observation of cervical mobility.

**Conclusion:**

The protocol may be a valuable tool to assess and monitor the care of patients with vestibular dysfunction, reducing healthcare costs for the therapist and patient and enabling the attendance of those with difficulties in traveling to the rehabilitation center or needing isolation.

## Introduction

Vestibular dysfunctions are characterized by dizziness, vertigo, vestibulo-visual symptoms, and changes in postural balance, 1 affecting 20 to 30% of the worldwide population throughout their lives, being more common in females and older people. 2 Patients with vestibular dysfunctions often avoid dangerous tasks or environments that may trigger the symptoms, 3 limiting their activities and social participation and resulting in social isolation and medical leave, negatively impacting their quality of life. 4 Also, psychiatric conditions (e.g., anxiety, panic disorder, and depression) are associated with vestibular dysfunctions. 5

Considering the limitations faced by patients with vestibular dysfunctions, a physical therapy assessment is essential to identify changes caused by these dysfunctions. This assessment is usually conducted in person; however, telerehabilitation has recently become a popular modality for care and assessments. This modality uses information and communication technologies in health services through remote meetings between health professionals and patients to assess, monitor, prevent, intervene, supervise, attend, and promote health education. 6

Previous research on remote assessments and interventions using telehealth showed that videoconferences had adequate measurement properties for assessing cognitive function, balance, and gait. 7 8 Also, telerehabilitation has been widely used for respiratory functions of patients with coronavirus disease 2019 (COVID-19), 9 neurological care after stroke, 10 and follow-up of older patients after hospital discharge. 11 In addition, it is cheaper and more viable than in-person rehabilitation 12 and may be equally effective, especially for improving physical function of patients with neurological and musculoskeletal dysfunctions. 13 14

Despite the variety of studies on telerehabilitation, research on its application in vestibular dysfunction is still scarce. Protocols for assessment and treatment of benign paroxysmal positional vertigo, 15 diagnosis of vestibular hypofunction, 16 a consensus for screening which patients should have urgent medical care or expedited outpatient care, 17 as well as guidelines on how teleconsultation can assist individuals with vertigo 18 were previously described. However, protocols for physical therapy assessment of the vestibular system considering functional factors and patient-centered results were not found. Therefore, this study aimed to propose a protocol for physical therapy assessment of central and peripheral vestibular dysfunctions using telerehabilitation. This protocol may help in the standardization of the tests to identify vestibular changes as well as monitoring assessments during the rehabilitation process.

## Methods

Although the present study did not have systematic review design, studies were summarized to structure the assessment protocol based on available scientific recommendations and clinical experience of physical therapists. A literature review on telerehabilitation in physical therapy was conducted in the PubMed and SciELO databases using the search terms *telehealth* , *telerehabilitation* , *vestibular disease* , *dizziness* , *vertigo* , and *postural balance* . Four physical therapists with at least four years of experience in vestibular rehabilitation discussed the information collected in the literature. Studies meeting the following criteria were included: 1) use of telerehabilitation or telerehabilitation as an assessment method for vestibular dysfunctions; 2) no restriction on publication year; and 3) no language restrictions. We excluded studies that involved in-person services, used telerehabilitation for individuals without vestibular dysfunction, performed device testing or studies focused on proving the effectiveness of vestibular rehabilitation exercises through telerehabilitation. The authors independently assessed titles, abstracts, and full articles for study inclusion.

Data was extracted from studies on bedside assessment in the otoneurology or vestibular physical therapy field using telerehabilitation, focusing on oculomotor, vestibular, and cervical evaluations, positional, static, and dynamic balance tests. The physical therapists also considered functional and clinical tests that are usually applied to assess vestibular dysfunction not previously described for remote modality. They discussed the requirements and methods to apply and adapt these tests for a remote assessment protocol and assessed the steps of the protocol to ensure feasibility of the suggested adjustments.

## Results

General orientations should be provided to patients before remote meetings (such as using a computer and/or smartphone with stable internet connection, trying to stay closer to the internet router, choosing a calm environment with adequate lighting and no distractions) to optimize the assessment of vestibular dysfunctions. Also, the presence of a companion during assessments is recommended for patient safety and adequate communication; 18 when necessary, they can film the assessments using the rear camera since many smartphones present low quality in the front camera. 15 Although these orientations are simple, some patients may be unable to undergo a remote assessment when the physical therapist considers that this modality may cause any risk to patients. For these patients, in-person assessments are recommended.

### Protocol for Remote Physical Therapy Assessments

The protocol can be applied synchronously or asynchronously. The synchronous modality includes live consultations between physical therapists and patients using tools provided by the health service (if available) or free web applications (such as Google Meet, Zoom, or WhatsApp). 17 The asynchronous modality can be used when connection problems hamper videoconferences, 17 in which the patient should send videos of the tests performed to the physical therapist for later analysis. In this modality, physical therapists must instruct patients previously by sending videos showing how to conduct the tests (e.g., oculomotor and postural balance tests) and guiding the ideal positioning of the camera to capture potential changes.

Initially, the protocol comprised sociodemographic (age, gender, marital status, education, and occupation) and clinical-functional aspects of patients (diagnosis, main complaint, history of the current dysfunction, comorbidities, use of medications, practice of physical exercise, smoking and dietary habits, alcohol consumption, sleep quality, presence of vestibular symptoms [dizziness, vertigo, vestibular-ocular symptoms, and postural instability], and symptoms intensity, duration, periodicity [daily, weekly, biweekly, or monthly], and time of onset). The final version of the protocol is shown in the Supplementary File .

### Assessment of Symptom Intensity

The numerical rating scale is an easy-to-apply and understand self-reported tool ranging from 0 (absence of symptoms) to 10 (most intense symptom), proposed to measure the intensity of dizziness or vertigo and postural instability. 19 Patients should be questioned on the intensity of symptoms at the time of assessment and in the previous week. Companions can help with understanding (if the physical therapist agrees) but should never answer questions for patients.

### Oculomotor Assessment

Regarding oculomotor assessment, some adaptations must be made to enable accurate evaluation. 17 The computer or smartphone should be placed on a stable surface to avoid false positives due to camera movement. Patients who wear glasses are instructed to remove them, except for the convergence test.

#### Eccentric Gaze

The physical therapist should share the screen containing a target and ask patients to bring their eyes closer to the camera and fix their gaze on the projected target. Ocular stability and symmetry and presence of spontaneous nystagmus should be observed. 20

#### Smooth Pursuit

Smooth pursuit is performed by following the path of a target displaced up to 30° in different directions only using the eyes. Patients should be instructed to remain with their heads still and use their thumbs or a pen to perform movements simulating the shape of the letter “H”, following the target path with the eyes. The intrinsic eye muscles should be observed, checking whether they follow the target at the same speed or semi-spontaneous nystagmus is present in the path covered. 21 The target should not be placed in front of the eyes since it would hamper the assessment by the physical therapist.

#### Saccades

Saccades are rapid and precise eye movements with static head. The repositioning movements of the fovea change the field of vision, and pauses in these movements are known as fixations. 22 Patients should be instructed to bring their eyes closer to the camera, and the physical therapist should provide random verbal commands for patients to look right, left, up, and down while maintaining the head still. The saccade speed and amplitude (hypo- or hypermetric) should be assessed, and its changes may imply cerebellar, brainstem, or extrinsic eye muscle damage or neuromuscular problems. 21

#### Vestibulo-Ocular Reflex Suppression

The vestibulo-ocular reflex (VOR) stabilizes vision during movements and head oscillations using compensatory eye movements in the opposite direction. The test aims to suppress this reflex, and patients maintain eyes and head fixed on the target and perform a synchronized movement of the head and target to the right and left or up and down. 23 24 In the adapted remote version of the test, patients should be instructed to hold the smartphone at the eye level with their arms extended. Next, patients should perform the same rotations as in the original test, maintaining their gaze fixed on the target. 17 The test is considered positive when patients present nystagmus or saccades or report blurred vision during the test, which might indicate central lesions. 21

#### Convergence Test

The convergence test assesses the shortest distance from the eyes to a target in which the image can be seen clearly. Patients should slowly bring an object or their thumbs towards the eyes and stop the movement when noticing diplopia or blurred vision. To assess symmetry in eye adduction remotely, patients should be positioned in front of the camera and perform convergence without covering the eyes with the camera. The final distance between the object and eyes should be assessed by patients positioned lateral to the camera, with the arm extended and the thumb at the eye level, and slowly approaching the thumb towards the eyes until noticing diplopia or suppression of one of the images. 21 The test indicates changes when patients report diplopia, blurred vision or suppression of one of the images at a distance greater than five centimeters from the eyes to the object. The cutoff point for the near point of convergence is seven centimeters. 25

#### Head Impulse Test

The test aims to identify possible unilateral or bilateral vestibular hypofunction through the VOR response by observing corrective saccades during rapid head movements in different planes. Originally, the test is performed with the examinator holding the patient's head at 30° cervical flexion and quickly moving it in the horizontal plane (10–20°), performing a cervical rotation while the patient keeps their eyes fixed on a target. The ipsilateral canal to the cervical movement is tested, while the VOR is contralateral. Hypofunction is considered positive when the eyes perform a corrective saccade at the end of head displacement to re-fixate the target in the field of vision. 21 24

In the remote test, the physical therapist should request active, quick, and short movements of the head to the right and left while looking at the camera to detect possible corrective saccades. 17 Companions should be also instructed to position the camera in front of the patients at eye level for a better view of their eyes during the test. An instructive video demonstrating the test can be sent to patients, if needed.

#### Test of Skew Deviation

Patients are instructed to gaze at a target, cover one eye with their hand, and wait five to ten seconds with a fixed gaze. The same process is conducted in the contralateral eye. The test is positive when a vertical skew is observed during the fixation on the target, which may indicate a central lesion. 20 The remote assessment of this test has a similar protocol, but patients should be instructed to position their faces close to the device's camera (target) for a better view of their eyes. 17

#### Optokinetic Reflex

The optokinetic reflex stabilizes the gaze during displacement of the visual field through “jumps,” or saccades, from one target to another, twice or three times per second. For the assessment, patients should be instructed to follow the moving stripes (horizontal and vertical) in a video with their eyes. The physical therapist shares the video, and patients must be positioned close to the camera on their device. The test is considered normal when the eyes follow the displacement of the target, causing a rapid return movement of the eye, like a “jump”. 21 24 26

### Positional Tests

Positional tests (such as Dix-Hallpike and Supine Roll tests to diagnose benign paroxysmal positional vertigo and otolith repositioning maneuvers) were not adapted in this protocol. However, another protocol was previously proposed to the remote modality to treat patients with benign paroxysmal positional vertigo and can be used with the present protocol. 15

### Cervical Assessment

The pain intensity in the cervical region at the assessment and previous week should be assessed using the numerical rating scale. Also, active cervical movements (flexion, extension, rotation and inclination to both sides, protrusion, and retraction) possible exacerbation of pain, and vestibular symptoms during movements should be assessed.

### Assessment of Standing Balance

Patients should be instructed to perform the tests in a wall corner to assess balance in different sensory conditions with a companion to ensure safety.

#### Modified Clinical Test of Sensory Interaction in Balance

In the in-person test, patients are instructed to maintain balance in four different sensory conditions (eyes open and feet together on the ground, eyes closed and feet together on the ground, eyes open and feet together on the foam, and eyes closed and feet together on the foam) to assess proprioception and contribution of vestibular input to static balance through changes of surface and removal of visual stimulus. 27 28

In the remote test, patients can use a cushion or pillow with at least 10 cm height and medium density to replace the foam. The test should be repeated 3 times, and the physical therapist should observe whether patients remain in positions for 30 seconds and present body oscillation in an anterior, posterior, or lateral direction and tendency to fall.

#### Single Leg Stance Test

Patients should be instructed to stand on the dominant lower limb with the contralateral knee flexed and without contact between the lower limbs. They should remain in a single leg stand for 30 seconds, with eyes open and closed. The test should be interrupted if the patient presents a tendency to fall or open their eyes (when performed with closed eyes). 29

### Assessment of Dynamic Balance

Gait is assessed using the Dynamic Gait Index for patients with vestibular dysfunction. 30 31 Although it has eight tasks, only the gait on a flat surface, gait with horizontal head turns, and gait with vertical head turns are suggested due to the limitations of telerehabilitation and to ensure patient safety. Tasks should be performed close to a wall and in the presence of a companion for the entire route. The score for each task ranges from 0 (severe impairment) to 3 (normal balance). 32

### Assessment of Quality of Life

The influence of dizziness on quality of life is assessed using the Dizziness Handicap Inventory. 33 34 This self-reported tool is composed of 25 questions on the physical (7 questions), functional (9 questions), and emotional (9 questions) aspects of the patient, with 3 answer options: no (0 points), sometimes (2 points), and yes (4 points). The final score is the sum of each question and ranges from 0 to 100 points; high scores indicate the worst quality of life due to vestibular symptoms. Scores are classified as mild (0–30), moderate (31–60), and severe (61–100). 35

## Discussion

The present study aimed to propose a protocol for physical therapists to assess vestibular dysfunctions remotely. Virtual platforms for monitoring patients have been considered feasible and encouraged in the clinical practice of healthcare professionals. 36 Telerehabilitation is also proving to be useful in promoting accessibility to physical therapy treatment for patients living far from rehabilitation or specialized centers or with difficulty traveling. For example, a specialized center in a regional service from Quebec (Canada) implemented a rehabilitation program to facilitate access and monitoring and avoid excessive travel by rural patients with spinal cord or traumatic brain injuries. 37 A scoping review demonstrated that the use of smartphones and apps in the treatment of patients with vertigo is feasible and promotes high-quality care. 38

The adoption of telerehabilitation is progressing slowly since virtual platforms are still adapting to the clinical practice of physical therapy. Also, studies comparing remote and in-person care on their effectiveness and technical management for patients with vestibular dysfunctions are scarce. Technical problems, such as internet connection and camera quality, are also barriers to adhering to this modality. 39 In addition, videoconferencing tools must be carefully selected. To mitigate risks, systems with data encryption, minimum risk of invasion, and without public access or those provided by health services are recommended to ensure data safety for patients and physical therapists. 40

Although telerehabilitation has limitations, remote assessments can be accurate and detailed if tests are properly adapted. 41 Also, a well-conducted anamnesis may indicate a syndromic, topographic, and etiological diagnosis in most patients with vertigo. 42 The asynchronous support may also improve the accuracy and consistency of the oculomotor assessment to identify changes that may not be observed in a synchronous assessment due to technical difficulties. For this purpose, patients should be instructed to use a cell phone to record a video of their eyes and maintain them open during the tests. 16 17

A study in the United States using telerehabilitation to treat vestibular dysfunction 43 observed that 86% of the 159 involved physical therapists reported success in remote interventions and clinical improvement in patients. Although this study suggested that telerehabilitation should be included in clinical practice to benefit patients who cannot travel to a rehabilitation center, further research is needed to assess and quantify the clinical improvement of these patients. In line with the items of our proposed protocol, the physical therapists reported using the observation of cervical range of motion (86%), oculomotor tests, such as smooth pursuit (77%), saccades (74%), and VOR suppression (58%), and the Romberg test for balance (74%). Also, around half of the physical therapists reported performing positional tests, while the remaining expressed discomfort in conducting these tests remotely. 43

The literature describes a clinical strategy model for diagnosing vestibular hypofunction using telerehabilitation, with information on technical and environmental conditions needed to conduct the test and a diagnostic strategy consisting of ocular, otolith, and balance assessment, 16 a consensus for screening patients with acute vestibular syndrome, 17 as well as guidelines on how teleconsultation can assist individuals with vertigo. 18 However, the protocol proposed in the present study was specific for physical therapists and included assessment of the cervical region, which helps to identify musculoskeletal changes that may influence the perception of otoneurological symptoms. It also included an adaptation of oculomotor tests, assessment of static and gait balance, and a questionnaire to analyze the impact of dizziness on the quality of life.

Some limitations related to the use of this technology should be considered, including technical challenges such as unstable internet connections and poor video and audio quality. 17 Additionally, patient-related factors, such as advanced age, lower educational levels, and limited digital literacy, may influence or restrict their ability to effectively use the technology. 44 45 A systematic review highlighted that the presence of cognitive impairments may also act as a limiting factor. 46

To minimize these barriers, studies suggest simplifying digital health tools and clearly demonstrating their benefits, using pictograms, simple language, and short slogans to enhance accessibility. 47 48 As technology adoption increases, more individuals may gradually become able to use these interventions, 48 mainly when involving younger caregivers. 49 In addition, the remote modality may reduce the costs of health services and help patients living far from a rehabilitation center, unable to travel, or with contagious diseases. In this sense, developing a protocol for physical therapists to assess vestibular dysfunctions remotely may standardize the execution of tests and facilitate the monitoring assessments during the rehabilitation process.

The current study's design is an initial definition of guidelines for assessing, monitoring and treating vestibular dysfunctions using telerehabilitation in the physical therapy field. Therefore, further studies should estimate the measurement properties (reliability, internal consistency, and validity) of assessment protocols for patients with vestibular symptoms in the in-person and remote modalities to identify equivalence of methods and outcomes.

## Conclusion

Telerehabilitation emerged as a viable option for assessments and interventions, especially after the COVID-19 pandemic. Although telerehabilitation for vestibular dysfunctions may present limitations, it might expand clinical practice. The protocol proposed in this study may be useful for assessing and monitoring patients with vestibular dysfunction, reducing health costs for the patient and physical therapist and providing treatment for patients with difficulties in traveling or needing isolation. Therefore, further studies should assess the validity, reliability, and acceptability of this tool in different contexts.
