# Supplementary material for: Telerehabilitation and Physical Therapy: Proposal for a Therapeutic Assessment Applied to Vestibular Dysfunctions
Source: Int Arch Otorhinolaryngol. 2025 Oct 16;29(4):1–7. doi: 10.1055/s-0045-1810116 (PMC12530908; doi:10.1055/s-0045-1810116)
Supplement: Supplementary file 1 — Supplementary Material [file 10-1055-s-0045-1810116-s241877.pdf]

Supplementary File

REMOTE THERAPEUTIC ASSESSMENT FORM FOR VESTIBULAR DYSFUNCTIONS

Physical therapist: ______________________ Assessment date: ____/____/_____

I – SOCIODEMOGRAPHIC DATA

Name: ____

Gender: ( ) Male ( ) Female Age: _______ Birth date: ____/____/_____

Address: ____

Phone: (____) _________________ Occupation:______________________________________

Marital status: ( ) Single ( ) Married or stable union ( ) Widowed ( ) Divorced

Educational level:

( ) Illiterate ( ) Middle school ( ) High school ( ) Higher education ( ) Postgraduate

II – CLINICAL-FUNCTIONAL DATA

Clinical diagnosis: ____

( ) Peripheral involvement ( ) Central involvement ( ) Mixed

Main complaint: ____

____

History of the current dysfunction:____

____

____

Comorbidities: ( ) Systemic Arterial Hypertension ( ) Diabetes Mellitus ( ) Other:

____

____

Use of medications:

____

____

Vestibular symptoms:

( ) Vertigo ( ) Dizziness ( ) Vestibular-ocular symptoms ( ) Postural instability

Duration of symptoms:______________

Periodicity of symptoms:

( ) Daily ( ) Weekly ( ) Biweekly ( ) Monthly Time of onset: ________

 © 2025. The Author(s).

Intensity:

0 1 2 3 4 5 6 7 8 9 10

Intensity of dizziness at the moment: _______

Intensity of dizziness in the last week: ______

Anthropometric measurements:

Height: ____cm Weight: _____kg BMI: ______ kg/m²

Lifestyle:

Alcohol consumption: ( ) Yes ( ) No ( ) Former Weekly:_______ How long?__________

Smoking: ( ) Yes ( ) No ( ) Former Weekly:_______ How long?__________

Dietary habits: ____

Sleep quality: ( ) Very good ( ) Fairly good ( ) Fairly bad ( )Very bad

Physical exercise:

Exercise practice: ( ) Yes ( ) No

Which? ( ) Weightlifting ( ) Running ( ) Swimming ( )
Other:____________________

Frequency: ( ) less than three times a week ( ) over three times a week ( ) Not applicable

III – OCULOMOTOR ASSESSMENT

TESTS	RESULTS
Eccentric gaze	( ) Normal<br>( ) Impaired
Smooth pursuit	( ) Normal<br>( ) Impaired
Saccades	( ) Normal<br>( ) Impaired
Vestibulo-ocular reflex suppression	( ) Normal<br>( ) Impaired
Convergence test<br>Distance: _______ cm	( ) Normal<br>( ) Impaired
Head impulse test	( ) Normal<br>( ) Impaired
Test of skew deviation	( ) Normal<br>( ) Impaired
Optokinetic reflex	( ) Normal<br>( ) Impaired

 © 2025. The Author(s).

IV – CERVICAL ASSESSMENT

Cervical pain: ( ) Yes ( ) No

Intensity:

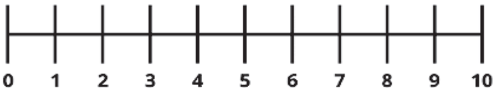

Pain intensity at the moment: ________

Pain intensity in the last week: _______

RANGE OF MOTION					
**Movement**	**ROM**	**Pain**	**Movement**	**ROM**	**Pain**
Flexion	( ) Normal<br>( ) Reduced	( ) Yes<br>( ) No	Extension	( ) Normal<br>( ) Reduced	( ) Yes<br>( ) No
Inclination R	( ) Normal<br>( ) Reduced	( ) Yes<br>( ) No	Inclination L	( ) Normal<br>( ) Reduced	( ) Yes<br>( ) No
Rotation R	( ) Normal<br>( ) Reduced	( ) Yes<br>( ) No	Rotation L	( ) Normal<br>( ) Reduced	( ) Yes<br>( ) No
Protrusion	( ) Normal<br>( ) Reduced	( ) Yes<br>( ) No	Retraction	( ) Normal<br>( ) Reduced	( ) Yes<br>( ) No
Exacerbation of any vestibular symptoms during movements:<br>____________________<br>____________________					

R – Right; L – Left.

V- ASSESSMENT OF STANDING BALANCE

MODIFIED CLINICAL TEST OF SENSORY INTERACTION IN BALANCE				
**Standing positions**	**Attempts (time in seconds)**			
	**1**	**2**	**3**	**Mean**
Eyes open and feet together on the ground				
Eyes closed and feet together on the ground				
Eyes open and feet together on the foam				
Eyes closed and feet together on the foam				

 © 2025. The Author(s).

SINGLE LEG STANCE TEST				
DOMINANT LOWER LIMB	POSITION			
	SINGLE LEG STAND WITH EYES OPEN		SINGLE LEG STAND WITH EYES CLOSED	
RIGHT	________ seconds	( ) Normal<br>( ) Impaired	________ seconds	( ) Normal<br>( ) Impaired
LEFT	________ seconds	( ) Normal<br>( ) Impaired	________ seconds	( ) Normal<br>( ) Impaired

 © 2025. The Author(s).
